# Special Considerations for Tympanoplasty Type I in the Oncological Pediatric Population: A Case-Control Study

**DOI:** 10.3389/fsurg.2022.844810

**Published:** 2022-03-08

**Authors:** Celine Richard, Emily Baker, Joshua Wood

**Affiliations:** ^1^Department of Otolaryngology, The University of Tennessee Health Science Center College of Medicine, Memphis, TN, United States; ^2^Division of Otolaryngology, St. Jude Children's Research Hospital, Memphis, TN, United States; ^3^The University of Tennessee Health Science Center College of Medicine, Memphis, TN, United States

**Keywords:** pediatric oncology, radiotherapy, chemotherapy, audiologic, otology, tympanoplasty, hearing loss

## Abstract

**Introduction:**

Although cutting-edges antineoplastic therapies increase survival in children with malignancies, the optimal surgical strategy to address associated comorbidities such as chronic tympanic membrane perforation is still poorly documented. The aim of this study is to evaluate the outcomes of type I tympanoplasty in pediatric cancer survivors who received chemo and/or radiotherapy to the skull and to identify potential associated risk factors.

**Methods:**

This case-control study included medical records review of oncologic patients (age <21) treated at the same Academic medical oncologic center between March 2015 and July 2021 and referred for conductive hearing loss and chronic tympanic membrane perforation. Patients and middle ear status-related variables were analyzed, and outcomes were compared with matched peers without any history of malignancies.

**Results:**

A total of seven pediatric cancer survivors and seven paired children without any history of malignancies were included in this report. The mean age at tympanoplasty type I surgery was 10.2 years (range = 4.3–19.9; median = 7.9 years) for the pediatric cancer survivors' group and 10.1 years (range = 5.5–19.2; median = 7.9 years) in the control group. Three pediatric cancer patients had received chemotherapy alone, one patient had radiotherapy to the skull base, and three patients had received chemoradiotherapy. On average, surgery was performed 3.9 years after chemo and/or radiotherapy termination, except for 1 patient for whom the tympanoplasty was performed during chemotherapy treatment. A retroauricular approach was used for one of the pediatric cancer patients, a transcanal approach was performed in one other and five patients benefited from an otoendoscopic approach. Tragal perichondrium with cartilage was used in most of the pediatric cancer survivor cases (four out seven cases) while xenograft (Biodesign) and Temporalis fascia without cartilage graft were used in five out of the seven control cases. Rate of tympanic membrane perforation recurrence was similar between groups (28.6%). Mean functional gain for air conduction Pure Tone Average (AC PTA) was 2.6 and 7.7 dB HL for the oncologic and control group, respectively. Mean postoperative air-bone gap (ABG) was 10.7 dB HL [median = 8.7; inter-quartile range (*IQR*) = 13.8] for the oncologic cohort and 10.1 dB HL (median = 10.7; *IQR* = 9.6) for the control group.

**Discussion:**

Chemo- and chemoradiotherapy to the skull are associated with damages to the inner and middle ear structures with secondary eustachian tube dysfunction and chronic middle ear effusion. Although healing abilities and immunological defenses are compromised as part of the expected effects of antineoplastic therapies, type I tympanoplasty can be safe and effective in this population. While different approaches may be considered, otoendoscopy showed excellent results with less morbidity in this vulnerable population.

## Introduction

With advances in chemotherapeutic agents and radiation modalities, survival prognosis has tremendously improved for children with malignancies (1). Besides these fantastic steps toward improved survival rate, the associated morbidities add an undesirable burden to the oncologic journey.

With a higher incidence of upper airways infections, anatomical peculiarities of the eustachian tube during childhood, adenoid hypertrophy, biofilm formation (2) among other factors, the pediatric population is at higher risks for chronic otitis media with effusion (OME). OME is the most common pediatric ear pathology, leading to a significant morbidity in this population. Although symptoms are usually unspecific, persistent OME causes hearing impairment, reportedly permanent in 2–35 per 10,000 (3). While controversies remain in the adult population as to the optimal management of radiation induced middle ear effusion, the cohort of children with malignancies follows the recommendations intended for the general pediatric population. General pediatric population guidelines recommend ventilation tube insertion in OME lasting ≥3 months, and/or with any associated impairments and/or with increased risk for speech and language development compromise (4).

Notwithstanding recent advances with targeted chemotherapy to specific molecular tumor profiles (5) and refinement of radiotherapy (6) to improve both effectiveness and safety, children with malignancies are at higher risks for middle ear pathologies compared to their healthy peers. Chemotherapy raises the risk for infection-related complications especially at the level of the upper respiratory tract (7). Radiotherapy can alter eustachian tube function and middle ear homeostasis (altered ciliary function, hyperreactivity in secretion) (8) while surgery may damage the parapharyngeal structures (9). Although the most prevalent, OME is not the only cause of conductive hearing loss in radiation-exposed children (10, 11). Others etiologies include chronic suppurative otitis media, tympanic membrane perforation (TMP), fibrotic changes of the middle ear mucosa, and/or ossicular necrosis (11). While OME is most frequent during radiation therapy, the mucosal damages to the middle ear (12, 13) associated with persistent ET dysfunction can lead to persistent OME after RT completion (11). Although ventilation tube placement will help with symptoms, the underlying cause may persist and compromise the outcomes of local procedures. One associated comorbidity in the pediatric oncologic population is represented by hearing loss that can be sensorineural (impairment at the level of the inner ear and/or subcortical-cortical structures), conductive (external and middle ear dysfunction), or mixed (both the sensorineural and conductive systems are affected). While radiation- and/or chemotherapy-induced damages to the inner ear are well-documented, current literature regarding the effects on middle ear and related surgeries is parse.

Therefore, we decided to conduct this case-control analysis to appraise the outcomes and peculiarities of type I tympanoplasty in the oncologic pediatric population.

## Materials and Methods

The St. Jude Children's Research Hospital Institutional Review Board and Le Bonheur Children's Hospital Institutional Review Board approved this retrospective study and its related protocol (**#** 21-0799). Patients with malignancies who receive chemo- or chemoradiotherapy and underwent surgical treatment for TMP between March 2015 and September 2021 were eligible for inclusion. Potential control peers were identified from the Pediatric Otolaryngology Head and Neck surgery database, spanning year 2012 to 2021. Both the oncologic and control groups were operated on by the same surgical team. Only children with history of type 1 tympanoplasty were included. In the present study, we referred to as type 1 tympanoplasty of any tympanic membrane reconstruction performed by lifting a formal tympanomeatal flap in a middle ear with normal ossicular chain status. Those diagnosed with previous history of tympanoplasty on the same ear, and/or history of cholesteatoma, and/or ossicular chain abnormality, and/or who underwent surgery after 21 years of age were excluded. Additionally, all patients were required to have a minimum of one postoperative clinical follow-up with pre- and postoperative audiometric data available to be included in the study. Each oncologic patient identified was matched with a control peer using the following criteria: type of surgery (type 1 tympanoplasty), age at surgery (±1.5 years), and size of tympanic perforation. Patient demographics, medical histories, and prior ear surgeries were recorded. Audiometric testing was performed by experienced audiologists using a pure-tone audiometer in a sound-proof booth, and thresholds were determined from 0.25 to 4 KHz. Hearing sensitivity within the speech frequencies was recorded according to the Academy of Otolaryngology-Head and Neck Surgery standards with four-tone air conduction (AC) pure-tone averages (PTA) obtained from AC thresholds collected at 0.5, 1, 2, and 3 kHz. Any missing values from the 3 kHz were replaced by the average value of the 2 and 4 kHz thresholds. The air-bone gap (ABG) was measured as the difference between air and bone conduction thresholds. The primary surgical outcome was recurrence rate for the oncological group and their matched peers. Surgical technique, including the approach, grafting material, and technique were collected.

Descriptive statistics are provided. Due to the skewness of the datasets, median, mean, and inter-quartile range (IQR) using quartile inclusive values are provided. Wilcoxon Rank Sum Test was applied for statistical analysis. Statistics were performed using R software version 4.0.4 9 [R Core Team (2013)]. R: A language and environment for statistical computing. R Foundation for Statistical Computing, Vienna, Austria. URL: http://www.R-project.org/). The *p*-values <0.05 were used as cut-off for statistical significance.

## Results

### Patients' Profile

We identified seven patients with malignancies who underwent type 1 tympanoplasty under 21 years of age. Among the 2,620 pediatric patients of non-oncology from the database who underwent tympanoplasty surgery, mostly were excluded based on the previous exclusion criteria. Seven non-oncologic patient controls were identified as best match based on their age at surgery, surgical technique, and operating surgical team. The mean age at tympanoplasty type I surgery was 10.2 (range = 4.3–19.9; median = 7.9 years) for the pediatric cancer survivors' group and 10.1 (range = 5.5–19.2; median = 7.9 years) in the control group. General characteristics and otologic history for the oncologic patients and their match are presented in Table 1. Etiology of the TMP was ventilation tube placement in five and in four of the oncologic and control patients, respectively (Table 1). All ventilation tubes placement occurred after primary tumor diagnosis and during oncologic treatment. The time between last set of ventilation tube placement and perforation diagnosis was not statistically different between the two groups (*W* = 3; *p* > 0.05), with a mean time of 1.6 ± 1.06 years (range = 0.6–3.6 years) and 5.4 ± 3.06 years (range = 1.2–8.3 years) for the oncologic and control group, respectively. The median time from perforation diagnosis to surgery was 14.7 months (range = 5.5–35.7 months; *IQR* = 14.1) for the oncologic cohort and 13.1 months (range = 4.6–20.9 months; *IQR* = 3.9) for the control group. Of note, neither underlying sinonasal infection nor recurrent upper airways infections were evidence in any of the patients included in either group.

**Table 1:** Patients' characteristics and otologic history.

	**Gender**	**Ethnicity**	**Primary malignancy**	**Ear surgery before cancer diagnosis**	**Previous ear surgery**	**Number of PET sets**	**Previous adenoidectomy**	**Associated syndrome**	**TMP etiology**
1	F	White, non-hispanic	Medulloblastoma	No	PET	3	No	No	*Tube-Related*
C #1	F	White	NA	NA	PET	1	Yes	No	*Tube-Related*
2	M	White, Hispanic	Left parapharyngeal RMS, with skull base and orbital extension	No	PET	1	No	No	*Tube-Related*
C #2	F	Unavailable	NA	NA	no	NA	No	No	Chronic Otitis Media
3	M	White, non-hispanic	B-cell ALL	No	PET	1	Yes 01/25/2019	No	*Tube-Related*
C #3	M	Unavailable	NA	NA	PET	5	Yes	No	*Tube-Related*
4	M	White, non-hispanic	Pre-B ALL	No	No	0	No	No	Unknown
C #4			NA	NA	PET	1	No	Ehlers Danlos	*Tube-Related*
5	F	Black	Optic pathway glioma	No	PET	1	No	NF1	*Tube-Related*
C #5	F	White, non-hispanic	NA	NA	no	NA	No	No	Draining AOM
6	M	White, non-hispanic	Neuroblastoma	No	PET and T tube	2	No	No	*Tube-Related*
C #6	F	Asian decent	NA	NA	PET	5	No	CLP	*Tube-Related*
7	M	Black and white	Chordoma	No	No	No	No	No	Unknown
C #7	F	White	NA	NA	PET	1	No	No	Post traumatic

*TMP, tympanic membrane perforation; C #, control patient; AOM, acute otitis media; CLP, cleft lip and palate*.

### Oncologic Treatments

The mean age at primary tumor diagnosis was 4.95 ± 4.04 years (range = 0.5–13.7 years; median = 3.5 years) and the time from diagnosis to treatment start was 18.6 weeks (range = 0.1–69.3 weeks; median = 6.7 weeks). Two children presented with a history of leukemia and received chemotherapy regimens including methotrexate. Cisplatin was part of the chemotherapy regimen for two cases (#1 and 6) and carboplatin for one case (#5). Chemoradiation was considered for three other patients. One patient presented with a chordoma and underwent surgery followed by radiotherapy to the clivus. The mean radiation dose to the cranium was 54.6 Gy and lasted from 26 to 61 days divided on 5 days weekly. Steroids was added to the drug regimens in two cases (#1 and 2) (Table 2).

**Table 2:** Lines of treatments and timeline to surgery.

**#**	**Primary**	**Age at tumor diagnosis (years)**	**End of chemotherapy-surgery (weeks)**	**End of radiotherapy-surgery (weeks)**	**First round** **chemotherapy**		**Second round** **chemotherapy**		**Third and fourth rounds chemotherapy**	**Radiotherapy**			
					**Regimen (high dose)**	**Duration (weeks)**	**Regimen (high dose)**	**Duration (weeks)**	**Regimen/duration**	**Field**	**Radiation dosage (Gy)**	**RT type**	**Duration (weeks)**
1	Medulloblastoma	2.5	97	89	Methotrexate, cisplatin, cyclophosphamide, and vincristine	15	Topotecan and cyclophosphamide	9	NA	Skull & Spine	54	Photon CSI	4
2	Left parapharyngeal RMS, extension to the skull base and orbit	5.25	65	120	Vincristine/irinotecan	63	NA	NA	NA	Left infratemporal fossa)	36	Proton	7
3	B-cell ALL	3.5	100	NA	Methotrexate and mercaptopurine	5	Mercaptopurine	125	NA	NA	NA	NA	NA
4	PreB ALL	6.8	63	NA	Methotrexate and mercaptopurine	128	NA	NA	NA	NA	NA	NA	NA
5	Optic pathway glioma	0.5	current chemotherapy	NA	Vincristine, carboplatin and temozolomide	83	Vinblastine	98	Selumetinib from 2017 to 2020 (147 weeks) and resumed in 2021	NA	NA	NA	NA
6	Neuroblastoma	2.4	825	860	Cyclophosphamide, doxorubicin, etoposide, cisplatin, melphalan, topotecan, tretinoin	72	NA	NA	NA	Abdomen	53	Photon CSI	6
7	Chordoma	13.7	NA	80	NA	NA	NA	NA	NA	Clivus	73.8	Proton	9

### Surgery and Timelines

Age at surgery did not significantly differ across groups (*W* = 25; *p* > 0.05), with 10.2 ± 5.3 years (range = 4.3–19.9 years; median = 7.9 years; *IQR* = 6.8 years) in the oncologic group and 10.1 ± 4.5 years (range = 5.5–19.2 years; median = 7.9 years; *IQR* = 5.2 years) in the control group. Time from the end of chemotherapy to surgery varied from 1.2 to 15.8 years (mean = 4.42 years; median = 1.86 years; *IQR* = 0.66 year) and time from the end of radiotherapy to surgery varied from 1.5 to 16.5 years (mean = 5.5 years; median = 2.01 year; *IQR* = 4.2 years), Table 2. Preoperative size of the TMP varied from 15 to 95% with a median of 30% for both groups (*IQR* = 20 for both groups, Table 3). All grafts were placed in underlay with a transcanal approach for three cases (two controls and one oncologic patient), a retroauricular approach for three others (two controls and one oncologic patient) and an otoendoscopic approach was used for the others (*N* = 8). In five out of the seven oncologic patients, cartilage was part of the graft materials whereas it was used in only two of the control patients, Table 3. All ears were dry at the time of surgery. However, two oncologic patient and two controls demonstrated inflammation of the middle ear mucosa during surgery (#5 and #6, C#3 and C#6).

**Table 3:** Otologic procedures.

	**Age at surgery (years)**	**Side**	**At surgery**					**Post-surgery**			
			**TMP location**	**TMP size (%)**	**Surgical approach**	**Surgical technique**	**Graft**	**TMP**	**TMP timing (months)**	**TMP location**	**TMP size (%)**
1	5.0	Left	Inferior	40	Transcanal	Underlay	TF	-	-	-	-
C #1	6.7	Left	Inferior posterior	30	Transcanal	Underlay	TF+Xenograft	-	-	-	-
2	7.9	Left	Subtotal	95	Retroauricular	Underlay	TF+Cartilage	-	-	-	-
C #2	7.9	Left	Inferior	80	Retroauricular	Underlay	TF	+	3.1	Inferior	10
3	7.9	Right	Anterior-inferior	20	Otoendoscopy	Underlay	Perichondrium+Cartilage	-	-	-	-
C #3	7.3	Left	Anterior-inferior	25	Retroauricular	Underlay	TF	-	-	-	-
4	10.7	Right	Anterior-inferior	40	Otoendoscopy	Underlay	Perichondrium+Cartilage	+	1.5	Central	10
C #4	11.2	Left	Central	40	Otoendoscopy	Underlay	Xenograft	+	1.7	Central	20
5	4.4	Left	Central-inferior	50	Otoendoscopy	Underlay	Perichondrium+Cartilage	+	5.3	Central	50
C #5	5.5	Left	Inferior	60	Otoendoscopy	Underlay	Perichondrium+Cartilage	-	-	-	-
6	19.9	Left	Anterior	30	Otoendoscopy	Underlay	Perichondrium+Cartilage	-	-	-	-
C #6	19.2	Right	Inferior	30	Otoendoscopy	Underlay	Perichondrium+Cartilage	-	-	-	-
7	15.8	Left	Anterior	15	Otoendoscopy	Underlay	Xenograft	-	-	-	-
C #7	13.2	Left	Posterior	30	Transcanal	Underlay	Xenograft	-	-	-	-

*C, control patient; TMP, tympanic membrane perforation; TMP timing (months), time from surgery to first diagnosis of TMP recurrence; TF, temporalis fascia*.

### Audiometric Status

The mean preoperative AC PTA was 24 dB HL (range = 11.2–41.2 dB; median = 22.5 dB; *IQR* = 12.2) for the oncologic group and 24.7 dB HL (range = 8.1–37.5 dB; median = 26.2 dB; *IQR* = 9.4) for the control group. Preoperative PTA was not significantly different between the oncologic and control groups (*W* = 11; *p* > 0.05).

**Figure 1:**
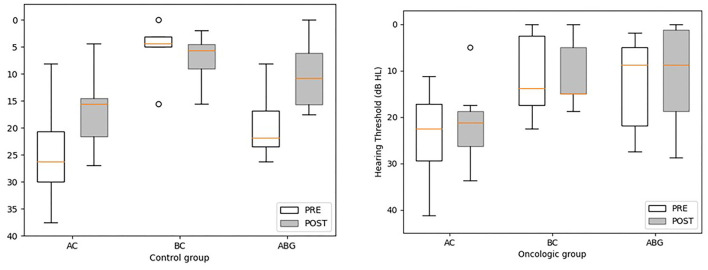
Pre-and post-treatment pure tone average thresholds in air conduction (AC), bone conduction (BC), and air-bone gap (ABG) for the oncologic and control groups. Statistical differences were analyzed using the Wilcoxon Rank Sum test. The postoperative BC PTA is missing for two children from the oncologic group (#5 and 7) and for 1 control patient (C#6). No values met the significance threshold of ≤ 0.05. A tendency was observed for the control group for postoperative AC PTA and ABG in comparison to preoperative values (*V* = 25 and *p* = 0.078 for AC PAT; *V* = 20, *p* = 0.06 for ABG).

Mean follow-up time after tympanoplasty was 17.4 months (range = 1.4–63.7 months; median = 6.97; *IQR* = 19.2) for the oncologic population and 22.8 months (range = 1.6–79.1 months; median = 5.7; *IQR* = 29.2) for controls. Bilateral chronic middle ear inflammation was reported in two oncologic patients (#1 and #5). However, the small sample size prevented us from drawing any conclusion or reaching any statistical significance. For both groups, the last AC PTA and ABG recorded had improved from preoperative data. The mean postoperative AC-PTA was 21.4 dB HL (range = 5–33.7 dB; median = 21.2 dB; *IQR* = 7.5) for the oncologic group and 17 dB HL (range = 4.4–26.9 dB; median = 15.6 dB; *IQR* = 7.05) for the control group. Mean functional gain for AC PTA was not significantly different between groups, with 2.6 and 7.7 dB HL for the oncologic and control group, respectively (*W* = 30; *p* > 0.05). Mean postoperative ABG was 10.7 dB HL (median = 8.7; *IQR* = 13.8) for the oncologic cohort and 10.1 dB HL (median = 10.7; *IQR* = 9.6) for the control group, Figure 1. The mean ABG functional gain for the oncologic group was not significantly different from the control group with 2.4 and 9.5 dB, respectively (W = 22; *p* > 0.05). Adherence to national cisplatin ototoxicity monitoring guidelines were observed in this study with serial pre-, per-, and post-treatment audiograms. However, children from the oncologic group who were not at risk for chemo-induced hearing loss had hearing monitoring during antineoplastic therapy but not always immediately after completion. For instance, #2 had his preoperative audiogram during chemotherapy that revealed an ABG = 1.9 dB HL, and a postoperative ABG of 15 dB HL with an AC PTA of 30 dB HL with a healed eardrum. Patient #5 was diagnosed with neurofibromatosis type I, enlargement of the brainstem, and bilateral optic pathway glioma. Although the patient's preoperative AC PTA was 22.5 dB HL, delays in conduction patterns were observed on the auditory brainstem responses and the patient had been fitted with hearing aids at 4 years of age and was receiving early speech and language therapy. For this patient, the surgery aimed at assisting with hearing aid adaptation by providing a dry ear, limiting the impact of chronic infection, and hearing loss on speech and language development and the quality of academic activities.

Four out of the seven oncologic patients presented with high-frequencies sensorineural hearing loss and an AC threshold ≥ 55 dB HL at 4 KHz (range 45–80 dB HL; mean = 58.75 dB HL). The sensorineural component of hearing loss was cisplatin-induced for two patients (#1 and 6) and radiation-induced for one (#2) who received radiation to the ipsilateral infratemporal fossa.

No difference was noted in PTA outcomes between the oncologic and control group at postoperative follow-up.

### Complications

No graft lateralization, blunting nor cholesteatoma was reported during follow-up. One oncologic patient #1 presented with a small retraction pocket anterior to the malleus for which the team elected for close monitoring. For this patient, temporalis fascia was the material graft used. Two patients from each group presented with a recurrent TMP (28.6%, Table 3). Recurrence time ranged from 1.5 to 5.3 months for the oncologic group vs. 1.7–3.1 months for the control group. For both oncologic patients, a cartilage was used during surgery for additional reinforcement. Patient #4 had his surgery 1.2 year after treatment completion and did not feature any specific signs of inflammation during surgery. Patient #5 was referred for tympanoplasty at an early age (4.4 years old) due to concern with chronic otorrhea and hearing aid adjustment. The team elected for tympanoplasty with a cartilage graft to provide an additional layer resistant to negative middle ear pressures (Table 2). She presented with a recurrent tympanic membrane perforation that failed two subsequent tympanoplasties.

### Postoperative Changes in Middle Ear Status

Patients #2 and #5 had an episode of postoperative middle ear effusion and patient #1 presented with recurrent episodes of postoperative left maxillary sinusitis with left middle ear effusion noted on serial control imaging for his primary tumor (left parapharyngeal rhabdomyosarcoma).

## Discussion

Type I tympanoplasty is nowadays a well-described surgery providing children with an improved hearing and dry ear. However, the oncologic population raises new challenges with a drug- and/or radiation-induced middle ear homeostasis disruption, delayed healing, and immune-system compromise. This is the first pediatric case-control study focusing on the potential factors affecting hearing and surgical outcomes in the oncologic population. This retrospective case series with matched controls provide a review and analysis of our experience with this vulnerable population. To our knowledge, no previous study has compared graft success and audiometric outcomes in this subset of patients.

The generally accepted definition of success encompasses the graft integrity and postoperative gain of more than 10 dB and neither OME recurrence nor atelectasis (14). Overall, surgical and hearing outcomes observed in this study did not significantly differ from the controls and from the general litterature (15–22). While comparing to the generally restrictive criteria (closure of the tympanic perforation with ABG ≤ 20 dB and an aerated middle space), four out of seven oncologic patients (57.1%) had a successful type I tympanoplasty, a rate slightly inferior to the report from Isaacson and Melaku (23). However, given the peculiarities of the presently reported oncologic population, and the disparities in age, TMP size and location, these criteria are difficult to apply to this study. When focusing on the rate of graft uptake, the oncologic population had the same rate as the control group (71.4%), but inferior to the mean weighted closure rate reported for pediatric tympanoplasty was 83.4% (24).

One of the main concerns when dealing with the oncologic population is to assess the optimized timing of surgery. The potential role of age as a prognosis factor of success is still subject to controversies (14, 25–27). However, the erratic eustachian tube function coupled with immunological immaturity of early childhood is one of the arguments for some teams justifying to delay tympanoplasty until 6 years of age in the general population (14). Although limited in size, no effect of age was observed in our oncologic cohort. Beyond the hearing improvement, the goal of type I tympanoplasty in children with malignancies is improve their quality of life by limiting the impact of the associated comorbidities. Type I tympanoplasty is also intended to help control otorrhea and assist with hearing aid fitting (11, 28). Early postoperative graft failure, within the first 3 months, is most commonly secondary to inadequate graft positioning, postoperative infection or pressure-related incident (i.e., early postoperative blowing) (29). However, a delayed failure (>3 months) is in most cases secondary to an underlying middle ear pathology. Effects of chemotherapy on the middle ear can be mediated through different ways. The pre-clinical studies showed the negative impact of chemotherapeutic agents on the immunologic status and on the wound healing process (30). The chemo-induced immune deficiency disrupts the middle ear homeostasis which is exposed higher risks for local infections (7), while its effects on cell division will impede fibroblasts proliferation (31), and subsequently impacts the course of TM healing. Among the different chemotherapies reported, vinblastine an alkaloid chemotherapeutic agent has been shown to affect microtubules affects tumors by impeding their cellular migration (32). Another concern for our oncologic population was the potential impact of the different chemotherapy regimens on wound healing, among which methotrexate has been well-reported (30). Although the limited number of patients prevents us from formulating any conclusions, both of our oncologic cases were either in an ongoing- or early post-chemotherapy phase.

Another factor that may jeopardize of graft uptake in the oncologic population is radiation. The effects of cranial radiation are reportedly notable with 82.5% of patient presenting with abnormal eustachian tube function and related middle ear dysfunction and with conductive hearing loss in one-third of patients (33, 34). In response to radiation, the TM thickens (35), middle ear mucosa undergoes edematous process with impaired gas exchanges, eustachian tube dysfunction resulting in a negative pressure and subsequent middle ear effusion (36). Radiation to the skull induces damages to the osteocytes and blood supply (37), and triggers repetitive inflammatory responses (38). All these changes are usually transient, lasting a couple of months (33). The timeline in recovery may explain the high rate of success observed for our radiated patients for whom the procedure was at distance from treatment completion (≥80 weeks). Two oncologic patients and two controls demonstrated inflammation of the middle ear mucosa during surgery (#5 and #6, C#3 and C#6), of whom only patients #5 presented with a postoperative effusion. The two other patients presenting with postoperative middle ear effusion (#1 and #2) were not reported with an inflamed middle ear mucosa at the time of the surgery. Based on our limited oncologic cohort, we cannot draw any conclusions whether a therapeutic mastoidectomy should be performed in case of inflamed middle ear status. However, based on the literature in non-oncologic patients, performing a therapeutic mastoidectomy does not improve the outcomes in patients with chronic otitis media (39). Moreover, in case of oncologic patients, their altered wound healing processes could increase the mastoidectomy-related morbidity.

Although **t**emporalis fascia is easily accessible and reliable as a graft material, the peculiarities of the oncologic middle ear supported the surgeon's choice of adding an extra layer of support with cartilage for most of the oncologic cases with a TMP ≥ 20% which is more restrictive than in the 50% reported in the literature (40). One exception was patient #1 for whom no cartilage was used for extra resistance. This patient presented with a retraction pocket within 3 months post-surgery. This finding corroborates previous report on the disrupted middle ear homeostasis secondary to antineoplastic therapies, placing the patient at higher risk for retraction pocket when considering temporalis fascia without any other support material (29). Another concern in our population is whether the radiated cartilage is an adequate graft material. Radiation-induced changes to the cartilage have been poorly studied. Although no graft failure was observed in our radiated patients, observations of scant cartilage matrix with decreased number of viable chondrocytes have been reported (41). When considering auditory outcomes, cartilage addition (0.5–1 mm width) when well-positioned without any direct contact with the sulcus has shown to have minimal impact on sound transmission (42–44). Although not significant, the control group tended to have better hearing outcomes (mean AC PTA and ABG functional gains) than the patients of oncology. Such results may be influenced by various factors among which the recurrence of middle ear effusion and/or the use of cartilage grafts. Surgical approach to the middle ear may vary, with a recent trend toward the use of otoendoscopes with an overall endoscopic success rate of 86.5% in the literature, increasing with the addition of a concurrent cartilage graft. The endoscopic tympanoplasty technique was refined and established itself as a recognized minimally invasive approach that limits the impact on the external auditory canal skin while providing an excellent view for graft positioning. It allows for better visualization in cases with tortuous bony canal or bony overhangs thus minimizing the rate of canalplasty (45–47) and avoiding its additional burden to a radiated bone. Endoscopic approach provides similar results to a microscopic approach on cochlear function, whether graft material is considered (48). The only limitation to endoscopic surgery is the ability of the surgeon with one-handed procedures and the need for an endoscope holders by some surgeons that may increase the exposure time of the middle ear to high temperature (49). Whether to choose an endoscopic or microscopic route, the postauricular approach, is more a matter of TMP size and surgeon's preference and training. In our experience, a more minimally invasive approach should be considered in pediatric oncologic patients presenting with a TMP <50%. By avoiding the need for a postauricular approach and canalplasty, the endoscopic approach allows for shorter operative times (46) that are advantageous in children especially in case of malignancies.

Given the substantial risk for TMP recurrence and the associated morbidity and impact of revision surgery, we believe reporting case in the specific subset of pediatric oncologic patients is critical. Although definitive conclusions are difficult to draw regarding the success rate of functional otologic surgery following chemo- or chemoradiotherapy; based on our institutional experience, type I tympanoplasty appeared to be safe and effective for more than half of the patients of oncology. There is a need for more reports in the oncologic population in order to better counsel patients and families. The clinician needs to be counseled on the possibility of TMP recurrence and the need for close long-term follow-up. However, we do believe that this functional surgery can improve their quality of life. Collecting further data will provide support for clinicians to discuss strategic choice in terms of timing, approaches, and graft material choices.

## Limitations

The main limitation of this case-control study is its limited sample size. Over quantity, we elected for rigorous inclusion criteria in order to better evaluate the probability of success of the therapeutic intervention. To ensure reproducibility of techniques, we only included surgery performed by the team of surgeons and excluded previous cases for which surgical technique may have varied causing additional bias to the outcome's evaluation. Moreover, oncological cases with a history of type 1 tympanoplasty are rare and poorly documented. Unfortunately, such drastic criteria in such a limited cohort prevented us from matching all patients for the type of graft and surgical approach. Another limitation of this study is the use of cartilage graft for five oncologic patients, which may have prevented an adequate postoperative evaluation of the middle ear status, with potential missed middle ear effusions.

## Conclusion

Adequate timing and optimized strategies may improve the surgical outcomes in this population. This study provides the pediatric otolaryngologist with an insight to quantify the probability of success for an oncologic patient and material to discuss the intervention with a patient and its family members.

## Data Availability Statement

The raw data supporting the conclusions of this article will be made available by the authors, without undue reservation.

## Ethics Statement

The studies involving human participants were reviewed and approved by the St. Jude Children's Research Hospital Institutional Review Board and Le Bonheur Children's Hospital Institutional Review Board approved this retrospective study and its related protocol (# 21-0799). Written informed consent to participate in this study was provided by the participants' legal guardian/next of kin.

## Author Contributions

JW and CR: study conception and design. EB, CR, and JW: data collection, analysis and interpretation of results, and draft manuscript preparation. All authors reviewed the results and approved the final version of the manuscript.

## Conflict of Interest

The authors declare that the research was conducted in the absence of any commercial or financial relationships that could be construed as a potential conflict of interest.

## Publisher's Note

All claims expressed in this article are solely those of the authors and do not necessarily represent those of their affiliated organizations, or those of the publisher, the editors and the reviewers. Any product that may be evaluated in this article, or claim that may be made by its manufacturer, is not guaranteed or endorsed by the publisher.
